# The first-generation *Daphnia magna *linkage map

**DOI:** 10.1186/1471-2164-11-508

**Published:** 2010-09-22

**Authors:** Jarkko Routtu, Bastiaan Jansen, Isabelle Colson, Luc De Meester, Dieter Ebert

**Affiliations:** 1 Zoologisches Institut, Evolutionsbiologie, Universität Basel, Vesalgasse 1, 4051 Basel, Switzerland; 2 Katholieke Universiteit Leuven, Charles de Bériotstraat 32, 3000 Leuven, Belgium; 3 Bangor University, Bangor LL57 2DG, UK

## Abstract

**Background:**

*Daphnia magna *is a well-established model species in ecotoxicology, ecology and evolution. Several new genomics tools are presently under development for this species; among them, a linkage map is a first requirement for estimating the genetic background of phenotypic traits in quantitative trait loci (QTL) studies and is also very useful in assembling the genome. It also enables comparative studies between *D. magna *and *D. pulex*, for which a linkage map already exists.

**Results:**

Here we describe the first genetic linkage map of *D. magna*. We generated 214 F2 (intercross) clonal lines as the foundation of the linkage analysis. The linkage map itself is based on 109 microsatellite markers, which produced ten major linkage groups ranging in size from 31.1 cM to 288.5 cM. The total size of this linkage map extends to 1211.6 Kosambi cM, and the average interval for the markers within linkage groups is 15.1 cM. The F2 clones can be used to map QTLs for traits that differ between the parental clones. We successfully mapped the location of two loci with infertility alleles, one inherited from the paternal clone (Iinb1) and the other from the maternal clone (Xinb3).

**Conclusions:**

The *D. magna *linkage map presented here provides extensive coverage of the genome and a given density of markers that enable us to detect QTLs of moderate to strong effects. It is similar in size to the linkage map of *D. pulex*.

## Background

*Daphnia magna *has been used in biological research since the 18th century [1]. At the beginning of 2010, a search for *Daphnia *in ISI Web of Knowledge^sm ^returned over 12,600 results starting from year 1900 [2]. *D. magna *is a target of many studies in evolution, ecology and ecotoxicology [3-5]. As a species, it is a relatively large (up to 5 mm), widespread and easy to maintain. Normally *D. magna *reproduces asexually, but it is possible to trigger sexual reproduction under controlled conditions, which is less easy in other members of the *Daphnia *genus. *D. magna *is also an ecologically important species in freshwater environments as it is a key grazer of algae while also being the preferred prey of fish [6]. It is widespread in the northern hemisphere, especially in the Palearctic [7]. Moreover, the taxonomy of *D. magna *is relatively straightforward compared to that of the *D. pulex *and *D. longispina *species complexes [8]. An advantage of *D. magna *over *D. pulex *is the relative ease of triggering sexual reproduction and hatching dormant eggs. We are rapidly acquiring new genomics tools for *D. magna *such as the genome project which all can be found in *Daphnia *Genomics Consortium webpage [9]. These attributes make this species a very desirable model organism for genetic studies.

The *D. pulex *linkage map is the first and most important reference point in comparing the genetic architecture of *D. magna*. These two species resemble each other phenotypically and share similar broad ecological niches. The divergence between *D. pulex *and *D. magna *had been estimated from mtDNA sequences to be about 200 MYA [10]. However, recent estimates of divergence time between these species, measured using nuclear genes, is reduced to 7.6 - 15.6 MYA depending on parameters [11].

*D. magna *is part of the sub-genus *Ctenodaphnia*, which have been reported to have 10 chromosomes (2n = 20) [12]. *D. pulex *(subgenus *Daphnia *s.s.) has 12 chromosomes, and its genetic map revealed 12 linkage groups (2n = 24) [13]. Chromosomes in *Daphnia *are small and contracted; thus, karyotypes are difficult to determine [12]. *D. magna *has no sex-chromosomes because sex determination in *Daphnia *is environmentally induced, so that the same genotype can be either male or female.

Dominant markers like AFLPs, RFLPs and RAPDs are fast and provide evidence of the overall genetic architecture, but do not effectively disentangle the exact genes responsible for trait differences [14]. Variable numbers of tandem repeats (VNTR) or microsatellite markers, on the other hand, are codominant markers and therefore provide more information. Here we report on a *D. magna *linkage study using microsatellite markers. The map is part of a larger QTL study aimed at detecting QTLs for various phenotypic traits. The map will also be instrumental in assembling the ongoing *D. magna *genome project.

The first objective of this study was to generate a linkage map of *D. magna *based on 109 VNTR marker loci. The second objective was to provide a test case of the *D. magna *linkage map's potential in QTL mapping. Combined with the published *D. pulex *linkage map, the linkage maps form a first-generation tool for comparative genomics of *Daphnia*.

## Results

### Linkage groups

**Figure 1:**
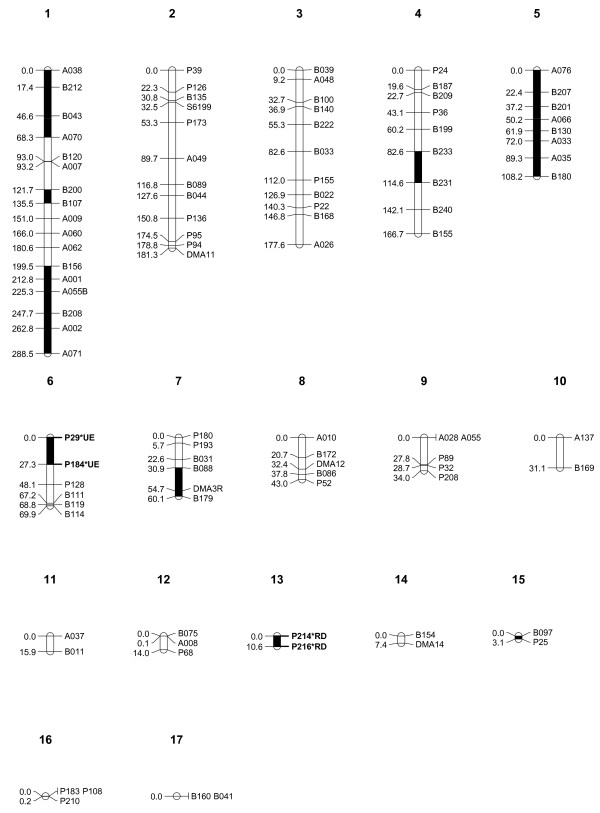
**Kosambi corrected linkage map of *D. magna***. Recombination distances in centiMorgans are on the left, and the name of the marker on the right. Locations of the two infertility alleles RD and UE are shown. Linkage groups 11 to 17 might be linked to the other groups if the marker density were higher.

**Figure 2:**
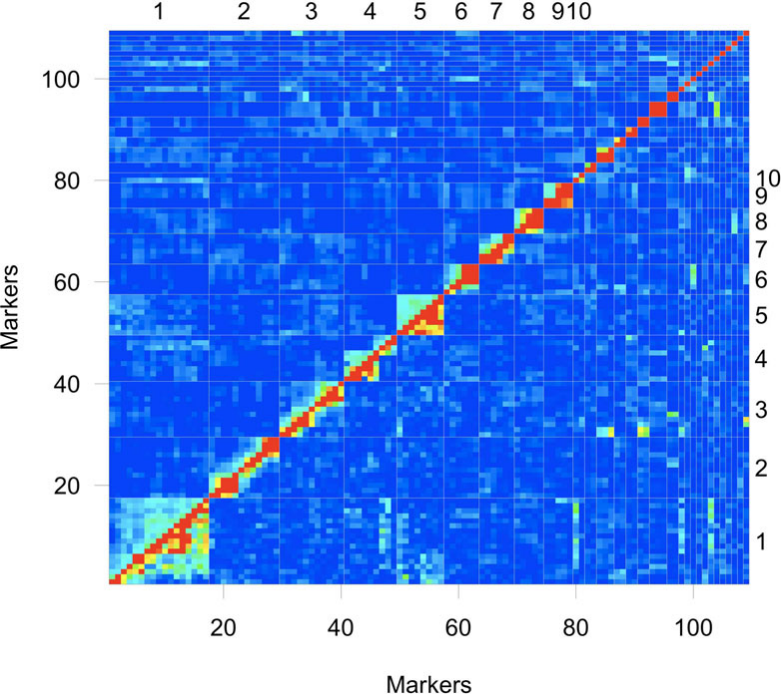
**Pair wise recombination fractions and LOD scores**. Marker (below and left side) and linkage group (above and right side) numbers are at identical positions as in the Figure 1. Above the diagonal; recombination fractions and below the diagonal; corresponding LOD scores. Red indicates high values and blue indicates low values. Green LOD scores are close to significant values.

The sum of Kosambi corrected map units was 1211.6 centiMorgans (cM) for the *D. magna *map (Table 1). The average interval for the markers in the linkage groups was 15.1 cM. The linkage analysis of the F2 panel resulted in 10 linkage groups of more than 30 cM with considerable variation in length (Figure 1). The largest linkage group was 288.5 cM, and the tenth largest linkage group was 31.1 cM. There were 12 unlinked markers and 7 small linkage groups (5 duplets and 2 triplets) ranging from 0-15.9 cM, indicating that our linkage map is not yet complete. The total length of unlinked small linkage groups was 51.2 cM, which is about 4% of the total map length. From all markers that were used, 74.3 percent showed linkage in the ten largest linkage groups. Many of the unlinked markers as well as the short duplets and triplets showed weak associations to the longer linkage groups (Figure 2). However, they could not be connected to the linkage groups with the critical recombination fraction value of 50 Haldane cM [15] and a LOD score of 3. The obtained Haldane distances were transformed to Kosambi [16] distances, which provide a better estimate of the real distances between markers by including recombination interference [17]. The EST VNTR markers are also directly mapped genes that are listed in Additional file 1 with their putative functions.

**Table 1:** Summary of the linkage groups (LG)

LG	Number of markers	Length Kosambi cM	Average spacing	Maximum spacing
1	17	288.5	18	29.2
2	12	181.3	16.5	36.4
3	11	177.6	17.8	30.8
4	9	166.7	20.8	32.0
5	8	108.2	15.5	22.4
6	6	69.9	14.0	27.3
7	6	60.1	12.0	23.9
8	5	43.0	10.7	20.7
9	5	34.0	8.5	27.8
10	2	31.1	31.1	31.1
11	2	15.9	15.9	15.9
12	3	14.0	7.0	13.9
13	2	10.6	10.6	10.6
14	2	7.4	7.4	7.4
15	2	3.1	3.1	3.1
16	3	0.2	0.1	0.2
17	2	0	0	0
18	1	0	NA	NA
19	1	0	NA	NA
20	1	0	NA	NA
21	1	0	NA	NA
22	1	0	NA	NA
23	1	0	NA	NA
24	1	0	NA	NA
25	1	0	NA	NA
26	1	0	NA	NA
27	1	0	NA	NA
28	1	0	NA	NA
29	1	0	NA	NA

overall	109	1211.6	15.1	36.4

### Transmission ratio distortion (TRD)

**Figure 3:**
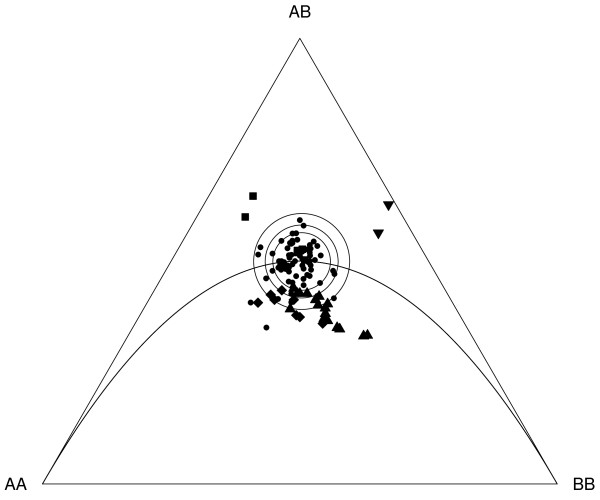
**Ternary plot (de Finetti diagram) of different marker genotypes**. Squares = linkage group 6; Diamonds = linkage group 5; Triangles tip up = linkage group 1; Triangles tip down = linkage group 13; Solid dots = all other genotypes. Large circles show the significant limits for χ^2^-test p-value. Symbols outside these circles show a significant deviation from the expected 1:2:1 ratio: from inside to outside P < 0.05, P < 0.01 and P < 0.001. The curved line indicates the expected Hardy-Weinberg equilibriums for all possible allele frequencies; below this line there are fewer heterozygotes than expected, and above it there are more than expected.

The overall number of heterozygote genotypes was 47.6 percent. The average frequency of heterozygotes did not deviate significantly from the expected distribution of 1:2:1 in F2 intercross design (χ^2 ^= 0.19, p = 0.67). However, individual markers had significant deviations of expected allele distribution (Figure 3, Additional file 2).

TRD was detected in 33% of the markers. Significant TRDs occurred in several clusters. The majority of the TRD was caused by a lack of heterozygotes (Figure 3), which was pronounced especially in linkage groups 1 and 5 (Figure 3). A lack of homozygotes was seen only in 4 markers belonging to linkage groups 6 and 13 (Figure 3).

### Infertility alleles

Iinb1 clone carried an infertility allele causing dwarfism, red coloration, and no egg production. We called this phenotype red dwarf (RD). Xinb3 clone carried an infertility allele causing an otherwise normal phenotype with the exception that asexual eggs would not hatch and no ephippia were produced--here called unviable eggs (UE). RD infertility allele mapped close to marker pair P214 (F_1, 312_= 163.1, p < 0.001) and P216 (F_1, 312_= 260.8, p < 0.001)(Additional file 2). Both these markers sit on the small linkage group 13 (Table 1, Additional file 2), which is not connected to the main groups. The UE allele mapped close to markers P128 (F_1, 312_= 12.3, p = 0.001), P184 (F_1, 312_= 86.9, p < 0.001) and P29 (F_1, 312_= 83.6, p < 0.001), which are part of linkage group 6 (Table 1, Additional file 2). The regions in which UE and RD alleles were located were not linked (see Figure 1). The infertility allele associations could be traced to the parental genotype/allele. RD infertility allele came from Iinb1 (the father), whereas UE infertility allele came from Xinb3 (the mother). We confirmed the origin of these two infertility alleles by selfing the parental clones, which resulted in approximately 25% of the infertility phenotypes in the selfed offspring of both parental lines. This is expected for a fully recessive allele being heterozygote in the parental type. The proportions of infertile individuals in F2 individuals were 73 RD, 78 UE and 210 fertile. These proportions follow the expected distribution of two recessive alleles in two unlinked loci. When the females showing infertility were excluded from the analysis, the χ^2 ^tests of TRD were P214 (χ^2 ^= 42.9, p < 0.001), P216 (χ^2 ^= 61.7, p < 0.001) for the UE type, and P29 (χ^2 ^= 31.0, p < 0.001) and P184 (χ^2 ^= 27.2, p < 0.001) (Additional file 2) for the RD type. But when sterile females (which we could not clone, but still used to assess some of their genotypes) were added to the analysis, the χ^2 ^values were strongly reduced and nonsignificant in the case of the UE type (P214 (χ^2 ^= 9.3, p = 0.009), P216 (χ^2 ^= 12.8, p = 0.002), P29 (χ^2 ^= 2.7, p = 0.25), P184 (χ^2 ^= 1.9, p = 0.39)). The four markers associated with the two infertility alleles are the four markers that show homozygote deficiency in Fig. 3 (squares and triangles tip down).

### Corrected linkage map length and coverage

The corrected linkage map length was 1519.9 cM for the ten largest linkage groups, which increased to 1692.5 cM when the unlinked smaller linkage groups were added. The coverage of the genome was 86.5%. The expected distance from a closest marker to a random gene was 7.7 cM when all the linkage groups and markers in them were used in the analysis.

### Genotyping error

Genotyping error was estimated to be 1.8 percent, which is slightly higher than the *D. pulex *linkage map estimate. To reduce the impact of potential errors, a small number of marker loci that produced ambiguous results were excluded from the linkage analysis.

## Discussion

The expected number of chromosomes in *D. magna *is 10 (= 1n) [12]. Our *D. magna *linkage map has ten linkage groups exceeding 30 cM. These are the best candidates for the ten linkage groups in the *D. magna *genome. However, the linkage map is a statistical approximation of the recombination pattern and thus not necessarily identical to the physical map.

The linkage map of *D. pulex *consists of 12 linkage groups [13]. The putative ten linkage groups in *D. magna *differ in some aspects from those of the *D. pulex *linkage groups. There is one large linkage group of 288.5 cM, which is approximately one third larger than the largest linkage group in the *D. pulex *linkage map. The lengths of the two genetic maps are rather close. The *D. pulex *Kosambi corrected linkage map spanned 1206 cM [13], whereas the *D. magna *extends 1211.6 Kosambi cM. Even if we exclude the unlinked small linkage groups, the putative linkage map of the ten chromosomes extends 1160.4 cM. We used 109 markers whereas for the *D. pulex *map 185 markers were used [13]. As a consequence, the average marker interval in the *D. pulex *linkage map is only 7 cM, as compared to rather larger 15 cM in the *D. magna *linkage map. However, in the *D. pulex *linkage map, a relatively large number (32%) of loci are at identical positions, possibly because the number of clonal lines (observed recombination events) used for the *D. pulex *linkage map is lower (129) compared to 214 for *D. magna *map. In the *D. magna *map, hardly any markers are at identical positions (Fig. 1).

A number of loci are not linked to the putative ten linkage groups of *D. magna*. This may be a result of incomplete linkage groups, which in turn suggests that the linkage groups extend further than in the present linkage map. There is also some indication of linkage between the unlinked loci themselves. The linkage map of *D. pulex *also resulted in some unlinked loci, but much less than in our study, which is likely to be explained by the smaller average marker distance [13].

We found several cases of TRD, i.e. deviations of the expected genotype distributions in the F2 cross (1:2:1). In two cases, TRD was caused by infertility alleles, which are heterozygote in the parental clones, with both alleles present in the F1 hybrid clone. When the genotypes of the sterile individuals are included into the χ^2^-test of linkage distortion, the χ^2 ^values are strongly reduced in linkage groups 6 and 13. However, the main cause of TRD in the *D. magna *linkage map is the lack of heterozygotes (Figure 3). Cristescu et al [13] found that 21% of their markers showed TRD, resulting in four regions that span about 30 to 70 cM. In contrast to the *D. magna *map, TRD in the *D. pulex *map was mainly caused by a lack of homozygotes [13]. *D. magna *has ten regions of TRD from 3.1 cM to 89 cM, covering 31% of the markers. An important factor influencing TRD in linkage studies is the genetic distance between the parental lines. Linkage studies on more distantly related parental strains report larger TRD proportions [18,19], with the strongest TRD in QTL studies between species [20]. This may be a consequence of losing synergistic epistatic interactions and resolved genetic conflicts between loci [21]. The parental lines of our panel came from *D. magna *populations that are strongly differentiated ecologically, about 1500 kilometers apart. It is likely that TRD caused by lack of heterozygotes in our study is influenced primarily by the large diversity of the parents, which makes their genomes partially incompatible.

## Conclusions

Based on the overall coverage of the *D. magna *genome by the linkage map described here and on the similarities of the *D. magna *and *D. pulex *linkage maps, we can infer that our *D. magna *linkage map captures the main elements of the genetic architecture of the *D. magna *genome. We used infertility alleles present in the parental lines to test that the F2 panel could successfully map the genetic basis of two recessive traits with simple genetics. This linkage map provides a starting point for targeted QTL studies and will be helpful in assembling the *D. magna *genome. It will need further refining using newly developed markers, and should in time help provide a true physical map of the *D. magna *genome.

## Methods

### The F2 intercross design

The parental clones of our F2 panel were Iinb1 (from Munich, Germany) and Xinb3 (Tvärminne, Finland). Xinb3 is the clone used for the ongoing *D. magna *genome project. Iinb1 was selfed for one generation, and Xinb3 was selfed for three generations to generate as much inbreeding as possible within the available time. *D. magna *clones from the Finnish coast self easily, whereas clones from Central Europe did not go easily through the sexual cycle. Male Iinb1 and female Xinb3 were used to produce a F1 hybrid clone. To maximize the range of traits that could be analyzed in the F2 panel, the parental clones were chosen based on divergent phenotypes on parasite resistance and behavioral traits, reflecting the very different ecology of the habitats they were isolated from. The resulting 214 viable F2 clones from the selfed F1 clone formed the basis for the linkage analysis.

### The markers

DNA from all clones was isolated with peqGOLD Tissue DNA Mini Kit (Peqlab, Erlangen, Germany). Each clone was genotyped for 109 markers: 29 of these were variable number of tandem repeats (VNTRs) developed from expressed sequence tags (ESTs) [22], and 80 were microsatellites (Jansen B, Geldof S, De Meester L Orsini, L: Isolation and characterization of microsatellite markers in the waterflea *Daphnia magna*, submitted). The 29 VNTR markers were multiplexed after PCR amplification to groups of non-overlapping marker alleles and analyzed in AB3130xl Sequencer (Applied Biosystems, Foster City, USA). PCR conditions were the same for all loci. An initial denaturation step of 4 minutes at 94°C was followed by 35 cycles of 94°C for 30 seconds, 52°C for 30 seconds, and 72°C for 30 seconds, followed by a final extension step of 72°C for 4 minutes. The 80 microsatellites were divided in 10 multiplexes. PCR's were done using Qiagen multiplex pcr kit with standard conditions and cycle times (Qiagen, Hilden, Germany). The initial activation step of one minute at 95°C was followed with 30 cycles of 94°C for 30 seconds, 56°C for 30 seconds, and 72°C 30 seconds followed by a final extension step of 60°C for 30 minutes. Putative functions were predicted for the EST VNTR markers (Additional file 1).

### Linkage analysis

The initial linkage analysis was done with the program MAPMAKER [23] using commands like; "group", "compare", and "map" for linkage groups of nine or less. For groups larger than nine markers, the command "try" was used to determine the exact position of a marker. The best order was examined with the command "lod table," where genetic distances and LOD scores are displayed as a matrix. Limiting thresholds in the map construction were minimum probability LOD 3 and maximum distance 50 Haldane centiMorgans. Once the markers were assigned to consensus linkage groups, the details of these groups were examined in R/qtl [24], which is an R [25,26] package for QTL analysis. Linkage analysis was not yet implemented in R/qtl, which was why the initial linkage analysis was done in MAPMAKER. However, we used the est.map() function in R/qtl to fine-tune the exact positions of the markers, introduce Kosambi correction and detect possible genotyping errors or reversed allele ordering.

### Mapping infertility alleles

Two sterility alleles inherited in the F2 generation as recessive alleles in two nonlinked loci were mapped using the single marker regression option in Windows QTL Cartographer version 2.5 [27]. We used single marker regression, which estimates the position and influence of infertility alleles' on the linkage analysis. We also determined from which parent the infertility alleles originated by examining the segregation pattern in the F2 genotypes and then selfing the parental clones. Fifty animals that hatched from resting eggs in the F2 panel construction of both infertility types were used in the linkage analysis, along with the 214 fertile F2 panel clones.

### Ternary plot (de Finetti diagram)

Ternary plot was used to visually infer the causes of significant TRD. We used R package HardyWeinberg to construct the plot [28]. The χ^2^-test significance levels 0.05, 0.01 and 0.001 were simulated in HWTernaryPlot() function and drawn separately on the graph.

### Corrected linkage map length and coverage

To estimate the corrected linkage map length and coverage, we followed the method of [21] and [29]. For each linkage group, two cM were added and then multiplied by *(m+1)/(m-1)*, where *m *is the number of markers in each linkage group. Linkage map coverage *c *was estimated as *c = 1 - e^-2dn/L^*, with *d *being the average distance of markers in the linkage map, *n *the number of markers, and *L *the length of the linkage map. Finally, we estimated the expected distance of a gene from the nearest random marker locus E (m) = L/(2*(n+1)) [17,30], where *m *is map units (cM) from the nearest marker, *L *is the linkage map length, and *n *is the number of markers.

### Genotyping error

Genotyping error was estimated from a random set of twice genotyped markers, 11 loci and 50 clones genotyped by two different people. The first set was genotyped with a ABI 310 Sequencer (Applied Biosystems, Foster City, USA), and the second set was genotyped with a AB3130xl Sequencer (Applied Biosystems, Foster City, USA).

## Authors' contributions

JR genotyped EST markers, analysed the data and wrote the manuscript. BJ developed and genotyped the microsatellite markers and contributed to data analysis. IC generated the F2 mapping population, developed and optimized EST markers, and initialized the infertility trait analysis. DE and LDM conceived the study, designed the F2 panel and edited the manuscript. The F2 panel was established in the Ebert lab. All authors read and approved the manuscript.

## Supplementary Material

Additional file 1 **EST VNTR markers predicted functions**. Name of the marker, EST id in wFleaBase, homolog in protein databases', E-value, predicted biological process and molecular function (excel file). Click here for file

Additional file 2 **Details of the marker loci**. Name, linkage group (LG), distance based on recombination fractions in Kosambi centiMorgans (cM), chi square test of linkage distortion (χ^2^), corresponding p value, accession number, forward primer, and reverse primer. The asteriks *** indicate markers that have significant associations with infertility phenotypes (excel file). Click here for file
